# SChLAP1 promotes prostate cancer development through interacting with EZH2 to mediate promoter methylation modification of multiple miRNAs of chromosome 5 with a DNMT3a-feedback loop

**DOI:** 10.1038/s41419-021-03455-8

**Published:** 2021-02-15

**Authors:** Kai Huang, Yuxin Tang

**Affiliations:** Department of Urology, The Third Xiangya Hospital, Central South University, Changsha, 410013 Hunan Province P.R. China

**Keywords:** Germ cell tumours, Germ cell tumours

## Abstract

This study aimed to investigate the mechanism of SChLAP1 (second chromosome locus associated with prostate-1) on microRNA expression in prostate cancer. Differential expression of lncRNAs and microRNA prostate cancer cells were predicted by informatics and confirmed by qRT-PCR. SChLAP1-interacting proteins were characterized by RNA pull-down combined with western blotting, which was verified using RIP and qPCR analysis. Then ChIP assay and DNA pull-down were used to validate the binding of DNMT3a and HEK27me3 with miRNA gene promoters. Target genes of miRNAs were bioinformatically predicted and validated by dual-luciferase reporter assays. The tumorigenicity of prostate cancer cells was assessed using the cancer cell line-based xenograft (CDX) model. We found that SChLAP1 expression was significantly elevated in prostate cancer tissues and cell lines, which was negatively correlated with miR-340 expression. SChLAP1 directly binds with EZH2 and repressed multiple miRNA expression on chromosome 5 including the miR-340-3p in prostate cancer cells through recruiting H3K27me3 to mediate promoter methylation modification of miR-340-5p/miR-143-3p/miR-145-5p to suppress gene transcription. Moreover, DNMT3a was one of the common target genes of miR-340-5p/miR-143-3p/miR-145-5p in prostate cancer cells. And SChLAP1/EZH2 could also promote prostate cancer tumor development via the interaction of microRNA-DNMT3a signaling pathways in xenograft nude mice. Altogether, our results suggest that SChLAP1 enhanced the proliferation, migration, and tumorigenicity of prostate cancer cells through interacting with EZH2 to recruit H2K27me3 and mediate promoter methylation modification of miR-340-5p/miR-143-3p/miR-145-5p with a DNMT3a-feedback loop.

## Introduction

Prostate cancer is one major human malignancy with high incidence, metastasis, and mortality rates due to asymptomatic feature at early stages, which is still listed as the second most common cancer in males and the fifth cause of cancer-related deaths all over the world^1,2^. Only in 2018, >1.2 million new prostate cancer cases were diagnosed, with a significantly elevated prevalence in the developed countries^2^. The initiation and progression of prostate cancer was known to be closely associated with multiple risk factor such as aging, ethnicity, accumulation of genetic factors, unhealthy dietary habits like saturated animal fats and red meats, alcohol consumption, as well as chronic prostatitis and other pathogenic conditions^2,3^. Also, prostate cancer development was shown to be mediated by the great alterations of signaling cascades including mutations in tumor-suppressor genes and oncogenes, cyclin-dependent kinase inhibitor-mediated cell cycle progression, the phosphatidylinositol 3-kinase/AKT (protein kinase B) pathway, and so on^1,3–5^. Over the past decades, surgeries, radiotherapy, androgen deprivation therapy, and chemotherapeutic regents were all prevalently applied for clinical management of prostate cancer, which, however, failed to substantially reduce prostate cancer incidence and improve patient survival^2,5,6^. The current high global incidence makes a call to fully elucidate the molecular events underlying prostate cancer pathogenesis.

MicroRNAs (miRNAs), as one large group of non-coding RNAs commonly composed of about 22 nucleotides, have been characterized as major regulators of prostate cancer development and metastasis due to their great capacities of post-transcriptionally modulating gene expression^7–9^. The development and metastasis of prostate cancer were closely linked with great alterations of miRNA profiles^7,8^. For instance, we previously discovered that miR-340 was capable of inhibiting the proliferation and metastasis of prostate cancer cells through directly targeting the mouse double minute 2 and p53 signaling pathways^10^. Also, miR-143 could also repress prostate cancer cell proliferation and migration as well as promote their sensitivities to chemo- and radio-therapies^11,12^. Similarly, miR-145 was also able to repress prostate cancer development via targeting the androgen receptor and regulating cancer stem cell activities^13,14^. Interestingly, the expression of above-mentioned tumor-suppressing miRNAs, which were encoded by genes localized on the No. 5 human chromosome, were all significantly inhibited in prostate cancer cells^10,11,13^. The molecular mechanisms underlying suppressed expression of these miRNAs would broaden our understanding of prostate cancer development.

Recent reports showed that the expression of cancer-related miRNAs could be substantially regulated by multiple epigenetic mechanisms, such as DNA methylation and histone methylation. For instance, the reduced miR-143/miR-145 expression in prostate cancer cells was closely associated with the hypermethylation of CpG dinucleotides in their promoter regions^12,15^, suggesting the significance of DNA methylation-induced miRNA suppression in prostate pathogenesis. Besides DNA methylation, histone modification was also reported to affect miRNA expression in cancer cells. EZH2 (Enhancer of Zeste Homolog 2) is a histone lysine methyltransferase that acts as one key component of the polycomb-repressive complex 2 complex to repress gene expression via catalyzing histone 3 lysine 27 tri-methylation (H3K27me3)^16^. More importantly, recent study showed that EZH2 activation contributed to the silencing of miRNA expression in prostate cancer cells, such as miR-101-3p and miR-138-5p^17^. However, the possible roles of EZH2-catalzyed H3K27me3 in regulating the expression of above-mentioned miR-340-5p/miR-143-3p/miR-145-5p encoded by chromosome 5 during prostate cancer development remains poorly understood.

Long non-coding RNAs (lncRNAs) are another type of non-coding RNAs larger than 200 nucleotides, which were also epigenetically implicated in prostate cancer pathogenesis^18,19^. For instance, the lncRNA MCM3AP-AS1 (MCM3AP antisense RNA 1) was recently reported to promote prostate cancer progression through activating the mitogen-activated protein kinase (MAPK) pathway^7^. LncRNAs have also been established as essential regulators of miRNA functioning in cancer development. The proliferation of prostate cancer cells could be enhanced by the lncRNA SNHG1 (Small Nucleolar RNA Host Gene 1) through its sponging with miR-199a-3p as a competing endogenous RNA^20^. However, little is known about the potential lncRNAs that could regulate miR-340/miR-143/miR-145 expression in prostate cancer pathogenesis. More importantly, the lncRNA-induced miRNA suppression in cancer progression was recently shown to be mediated by EZH2, which directly represses miR-214 expression in cervical cancer^21^. The lncRNAs capable of regulating EZH2-catalzyed H3K27me3 and its possible effects on miR-340/miR-143/miR-145 expression in prostate cancer cells deserve further investigations.

In this study, we aimed to investigate the lncRNA-mediated expressional regulation of miR-340/miR-143/miR-145 encoded by chromosome 5 in prostate cancer cells, as well as the molecular mechanisms and pathogenic roles in cancer development. The possible involvement of H3K27me3 catalyzed by EZH2 in lncRNA-induced miRNA suppression was also tested. We revealed that the lncRNA SChLAP1 (second chromosome locus associated with prostate-1) promotes prostate cancer development via interacting with EZH2 to promote H2K27me3 and regulate miR-340-5p/miR-143-3p/miR-145-5p expression. These investigations provided new insights into the epigenetic landscape of prostate cancer pathogenesis, which might serve as novel targets for cancer diagnosis and clinical treatment.

## Materials and methods

### Clinical samples

The prostate cancer tissues (tumor) and adjacent non-cancerous prostatic tissues (non-tumor) were collected from 30 patients who were diagnosed with prostate cancer and underwent surgical treatments in the Department of Urology of the Third Xiangya Hospital, Central South University (Changsha, Hunan province, China) from 2018 to 2019. The experimental procedures involving clinical samples were approved by the Ethics Committee of the Third Xiangya Hospital of Central South University, and written informed consent was obtained from each patient before surgery.

### Cell culture and treatments

The human prostate cancer cell lines 22RV1, DU145, LNcap, Vcap, and PC-3, as well as the human normal prostate epithelial cell line RWPE-1, were purchased from the Cell Bank of the Chinese Academy of Science (Shanghai, China). These cells were cultured in exactly matched basic medium containing 10% fetal bovine serum (Gibco) at 37 °C in standard humidified chambers with 5% CO_2_. Cell line identifies were confirmed by the short tandem repeat profiling method. To inhibit the pathways of methylation regulation, some inhibitors were used to treat prostate cancer cells with the indicated concentration and time. All these inhibitors were purchased from Selleck: GSK343 (S7164), gamma-Oryzanol (S3957), EED226 (S8496), CPI-455 HCl (S8287), and UNC1999 (S7165).

### Oligonucleotides, constructs, and cell transfection

The miRNA mimics and inhibitors of hsa-miR-340-5p, hsa-miR-145-5p, and hsa-miR-143-3p and their corresponding negative controls were synthesized by the GenePharma Company (Shanghai, China). DNMT3a wild-type (WT) and mutant (MUT) sequences, synthesized by the Sangon Biotech Company (Shanghai, China), were ligated onto the psicheck2 plasmid (Promega). For cell treatment, above oligonucleotides and recombinant plasmids were transfected into the prostate cancer cells using the lipofectamine 3000 reagent (Thermo Fishier Scientific, USA) following the manufacturer’s instructions.

### Recombinant lentivirus vectors and transfection

The lentivirus-mediated stable overexpression or knockdown of SChLAP1, EZH2, and DNMT3a in prostate cancer cells were carried out as previously introduced^18^. Briefly, the CDS sequences of SChLAP1, EZH2, and DNMT3a prepared by artificial synthesis, as well as the SChLAP1 short hairpin RNA (shRNA; 5’-GGGAGAGTCATCCAAGGAA-3’), EZH2 shRNA (5’-GCTAGGTTAATTGGGACCAAA-3’), and DNMT3a shRNA (5’-CCACCAGAAGAAGAGAAGAAT-3’), were separately ligated with the GV113 lentivirus vectors, which were then used to transfection into 293T cells. Lentiviral particles were collected from the culture supernatants at 48 h later after transfection. Followed by purification and titer detection, these special lentiviruses were then infected with prostate cancer cells as designated with appropriate multiplicity of infection.

### Bioinformatics

Expressional alterations of lncRNAs and miRNAs in prostate cancer patients were analyzed using the UALCAN website based on The Cancer Genome Atlas database (http://ualcan.path.uab.edu/) and the starBase platform (Pan-cancer module; http://starbase.sysu.edu.cn/). The interaction between lncRNA and protein was predicted by RNA-Protein Interaction Prediction (http://pridb.gdcb.iastate.edu/RPISeq/). The target genes of miR-340-5p, miR-143-3p, and miR-145-5p were predicted by the online software TargetScan 7.2 (http://www.targetscan.org/vert_72/).

### Quantitative reverse transcription (RT)-PCR

Total RNA samples were prepared from cell and tissue samples using the Trizol solution (15596018, Invitrogen, USA) according to the manufacturer’s instructions. RNA concentrations were determined by the NanoDrop 2000 instrument (Thermo Fishier Scientific). Subsequently, cDNA samples were synthesized by RT using the miScript II RT Kit (#218160; QIAGEN) following the manufacturer’s instructions. Finally, the expression of target genes was relatively quantitated by the real-time quantitative PCR (qPCR) method using the Bestar^TM^ qPCR MasterMix Kit (#2043; DBI) as instructed by the manufacturer. The standard 2^−△△Ct^ method was applied in this study for calculation of relative expression difference in each group sample. Glyceraldehyde 3-phosphate dehydrogenase and U6 were simultaneously detected as internal reference. Sequences of primers used for expressional quantitation are presented in Table 1.

**Table 1:** Primer sequences used for quantitative PCR assay.

Gene ID	Primer sequences (5’–3’)
PCGEM1 F	AGATGCACTGGGACTCAACG
PCGEM1 R	CCCTAGGAGTAGGCCTGTGT
CTBP1-AS F	GTGTGAGACCCTTGCTCACC
CTBP1-AS R	AGTGTACCCTTTCCACCCCT
SChLAP1 F	CAAGTCCTCAGGTGCCATCAA
SChLAP1 R	GGCACTTCTTCCCCAGTCAT
miR-340-5p RT	GTCGTATCCAGTGCAGGGTCCGAGGTATTCGCACTGGATACGAC AATCAG
miR-340-5p F	CGCGGCTTATAAAGCAATGAGA
miR-143-3p RT	GTCGTATCCAGTGCAGGGTCCGAGGTATTCGCACTGGATACGACTGAGCT
miR-143-3p F	GCGCTGAGATGAAGCACTGT
miR-145-5p RT	GTCGTATCCAGTGCAGGGTCCGAGGTATTCGCACTGGATACGACAGGGAT
miR-145-5p F	CGGTCCAGTTTTCCCAGGA
GAPDH F	CCAGGTGGTCTCCTCTGA
GAPDH R	GCTGTAGCCAAATCGTTGT
U6 F	CTCGCTTCGGCAGCACA
U6 R	AACGCTTCACGAATTTGCGT

### Western blotting

Total proteins from culture cells or tissues were prepared using the Total Protein Extraction Kit (#78503, Thermo Scientific, USA). Protein concentration was determined using the BCA Protein Assay Kit (Sangon Biotech, Shanghai, China) according to the manufacturer’s instructions. Fifty micrograms of total proteins were separated using sodium dodecyl sulfate–polyacrylamide gel electrophoresis, transferred onto polyvinylidene difluoride membranes, which were subsequently blocked with 5% bovine serum albumin, and incubated with diluted primary and secondary antibodies. Relative protein levels were finally quantitated by developing with the ECL Substrate Kit (#32109, Thermo Scientific, USA). β-Actin proteins were analyzed as the internal standard for normalization. The following primary antibodies were used in this study: EZH2 (Abcam, Cambridge, UK, ab227648), DNA methyltransferase 1 (DNMT1; Abcam, Cambridge, UK, ab92314), DNMT3a (Abcam, Cambridge, UK, ab227725), DNMT3b (Abcam, Cambridge, UK, ab227883), H3K27me3 (Abcam, Cambridge, UK, ab192985), and H3K4me3 (Abcam, Cambridge, UK, ab8580). Quantification of the protein expression was by the ImageJ software (National Institutes of Health).

### Tumor cell proliferation and migration analysis

The proliferation rates of prostate cancer cells were evaluated by the colony-formation assay following previous description^22^. Briefly, approximately 500 prostate cancer cells were cultured in each well of a 6-well plate at 37 °C, followed by constant culture under normal conditions for another 12 days. The cell clones formed in 6-well plates were subsequently stained with 0.5% crystal violet solution for 30 min and finally observed and photographed under light microscopy. The metastatic capacity of prostate cancer cells was detected using the Transwell system as previously introduced^23^. Briefly, prostate cancer cells were seeded in the upper chambers filled with serum-free Dulbecco’s modified Eagle’s medium while the lower chambers were added with complete medium. After culture for 24 h, prostate cancer cells in the lower chambers were stained with crystal violet and observed under microscopy. The cell count statistics was analyzed using the software ImageJ.

### RNA pull-down and western blot analysis

For characterization proteins that associated with SChLAP1, the RNA pull-down assay was performed using the Pierce™ Magnetic RNA-Protein Pull-Down Kit (#20164; Thermo Fishier Scientific) following the instruction manual. The target SChLAP1 was first labeled with RNA 3´ Desthiobiotinylation Kit (#20163; Thermo Fishier Scientific), which was then bound with the Streptavidin Magnetic Beads and incubated with the prostate cancer cell lysates for 45 min at 4 °C with gentle rotation. After washing, the RNA-binding proteins were eluted with 50 µL Elution Buffer and then validated by western blot analysis.

### RNA-immunoprecipitation (RIP) assay

The binding of EZH2 protein with SChLAP1 was verified in this study by the RIP assay using the EZ-Magna RIP™ RIP Kit (Merck, Germany) following the manufacturer’s instructions, in combination with qPCR method. Briefly, prostate cancer cells were lysed in RIP lysis buffer, incubated with magnetic beads containing antibodies specifically recognizing EZH2 proteins, and incubated with Proteinase K. Magnetic beads conjugated with antibodies targeting human IgG were used as the negative control. After washing with the washing buffer, RNA samples bound in magnetic beads were eluted and used as the templates for RT. The relative contents of SChLAP1 in elutes were analyzed by qPCR method.

### Chromatin immunoprecipitation (ChIP) and DNA pull-down analysis

The association of methylated regulatory proteins (EZH2/H3K27me3/DNMTs) with the promoter sequences of hsa-miR-340, hsa-miR-143, and hsa-miR-145 genes were validated by the ChIP method and DNA pull-down analysis. Briefly, DNA fragments were first prepared from prostate cancer cells assisted by sonication and collected by centrifugation at 13,000 × *g* for 10 min at 4 °C, which were then immunoprecipitated with antibodies targeting EZH2 protein or H2K27me3 (ChIP grade; Abcam). Subsequently, DNA fragment bound with the above antibodies was purified using 80 µL DNA-purifying slurry and detected by PCR method using primers targeting the promoter sequences of hsa-miR-340, hsa-miR-143, or hsa-miR-145. For DNA pull-down assay, probes, respectively, targeted with hsa-miR-340, hsa-miR-143, or hsa-miR-145 promoter were designed and labeled with desthiobiotin, and then bound with streptavidin-containing magnetic beads and in turn incubated with prostate cancer cell lysates. After washing with PBS for three times, the eluates were subjected to western blotting analysis for the target proteins.

### Dual-luciferase reporter assay

The binding of miRNAs with DNMT3a gene 3’ untranslated region (3’UTR) sequences were validated here by the dual-luciferase reporter assay using the Nano-Glo® Kit (Promega) following the producer’s instructions. Briefly, the WT or MUT plasmids containing the 3’UTR sequences of DNMT3a gene were co-transfected into the cellular tool HEK293 cell line with miRNA mimics as designated. Forty-eight hours of incubation after transfection, the luciferase activities in the cell lysate samples were measured using the GloMax® 20/20 luminometer.

### In vivo tumorigenesis

The cancer cell line-based xenograft (CDX) model was established to assess the tumorigenicity of prostate cancer cells. Twenty-five male nude mice (6 weeks old) were randomly divided into 5 groups (5 each). Briefly, DU145 cells (1.5 × 10^6^) stably expressing SChLAP1-shRNA or DNMT3a gene were introduced into the rear flank of nude mice by subcutaneous injection. Subsequently, the formation of tumor in mice were monitored for the following 30 days, and tumor sizes were recorded every 5 days. Finally, the nude mice were sacrificed after anesthesia and tumor tissues were collected and used for the following detection analysis. The Ethics Committee of the Third Xiangya Hospital of Central South University approved the study protocol for the use of experimental animals.

### Statistical analysis

Quantitative results obtained from at least three biological replicates were shown as mean ± standard deviation (SD) and analyzed using the SPSS 20.0 software. Significant differences between two or more groups were determined by Student *t* test or analysis of variance method, respectively, using *P* < 0.05 as the threshold.

## Results

### SChLAP1 repressed multiple miRNA expression on chromosome 5, including the miR-340-3p in prostate cancer cells

**Fig. 1:**
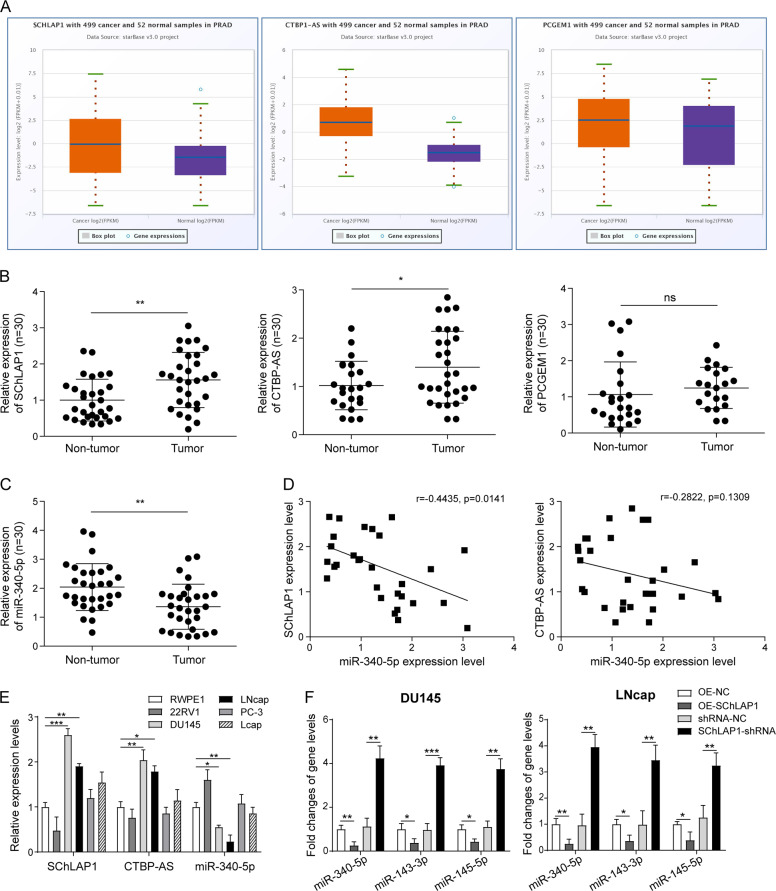
LncRNA SChLAP1 was significantly overexpressed in prostate cancer tissues and cells and was negatively correlated with miR-340-5p. **A** Bioinformatic analysis of lncRNA SChLAP1, CTBP1-AS, and PCGEM1 from TCGA database by the online software starBase v3.0. **B** QPCR analysis for the expression levels of lncRNA SChLAP1, CTBP1-AS, and PCGEM1 in 30 pair clinical specimens. Paired *T* test was used. *n* = 30, *P < 0.05, **P < 0.01. **C** QPCR analysis for the expression levels of miR-340-5p in 30 pair clinical specimens. **D** The expressive correlation analysis between SChLAP1/CTBP1-AS and miR-340-5p in the 30 pair clinical specimens. The correlation was measured by Pearson correlation analysis. **E** QPCR analysis for the differential expression levels of lncRNA SChLAP1, CTBP1-AS, and miR-340-5p in prostate cancer cell lines 22RV1, DU145, LNcap, PC-3, and Vcap compared to the normal prostatic epithelial cell line RWPE-1. **F** QPCR analysis for the expression levels of miR-340-5p, miR-143-3p, and miR-145-5p in DU145 and LNcap cells with SChLAP1 overexpression or knockdown. The results are presented as the mean ± SD. *P < 0.05, **P < 0.01, ***P < 0.001. SChLAP1 second chromosome locus associated with prostate-1, CTBP1-AS C-terminal binding protein 1 antisense, PCGEM1 prostate cancer gene expression marker 1, TCGA The Cancer Genome Atlas.

Our previous study has found that the expression of miR-340-5p were significantly downregulated and act as a tumor suppressor for prostate cancer in in vitro and in vivo experiments^23^. Based on the functional characteristics of lncRNA in regulating target gene expression through methylation, we speculated that the downregulation of miR-340-5p might be related to this mechanism. To verify this hypothesis, we performed a mass of bioinformatic analysis using online databases and software. First of all, we focused on three lncRNAs including SChLAP1, CTBP1-AS, and PCGEM1 (Fig. 1A) through further big data analysis of clinical samples. Then the expression levels of these lncRNAs were further verified in 30 pair clinical specimens by qPCR analysis, as well as the miR-340-5p. Expected results were obtained that SChLAP1 and CTBP1-AS were upregulated in prostate tumor tissues than that in the non-tumor tissues (Fig. 1B), while the miR-340-5p were inversely downregulated (Fig. 1C). The PCGEM1 level was also increased but not significant (Fig. 1B). Then the expressive correlation between SChLAP1/CTBP1-AS and miR-340-5p were further confirmed in the 30 pair clinical specimens. Results showed that there was a significant negative correlation between SChLAP1 and miR-340-5p, while the negative correlation between CTBP1-AS and miR-340-5p was not significant (Fig. 1D). Consistent results were confirmed in in vitro prostate cancer cell lines (Fig. 1E). In order to confirm the effects of SChLAP1 on the expression of miR-340-5p, qPCR was performed in DU145 and LNcap cells with SChLAP1 overexpression or knockdown, as well as several other miRNAs all located on the chromosome 5, such as the miR-145-5p and miR-143-3p. Results showed that the expression of miR-340-5p, miR-143-3p, and miR-145-5p in DU145 and LNcap cells were significantly reduced when SChLAP1 was overexpressed and greatly promoted when SChLAP1 was silenced (Fig. 1F). These above results indicated that the miR-340-5p, miR-143-3p, and miR-145-5p were inhibited by lncRNA SChLAP1 in prostate cancer.

### SChLAP1 interacted with EZH2 and DNMT3a in prostate cancer cells

**Fig. 2:**
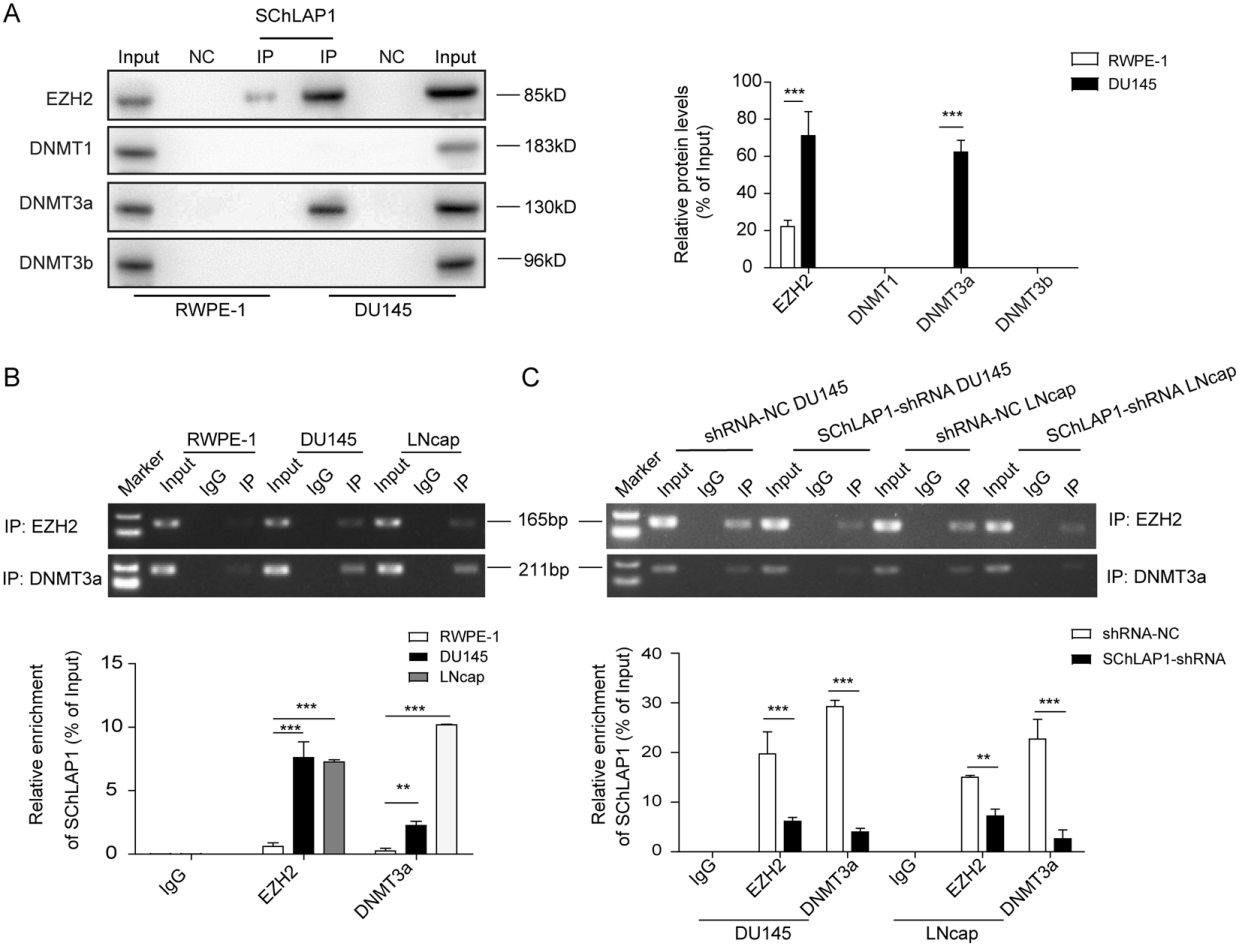
By interaction with EZH2, SChLAP1 repressed multiple miRNA expression on chromosome 5 including the miR-340-3p in prostate cancer cells. **A** RNA pull-down combined with western blotting analysis by lncRNA SChLAP1 probe for the interaction with EZH2 or DNMTs (DNMT1, DNMT3a, and DNMT3b) in RWPE-1 and DU145 cells. Negative control (NC): antisense RNA probe, IP: immunoprecipitation. **B** RIP assay combined with RT-qPCR analysis by EZH2 antibody to validate direct interaction between EZH2 and SChLAP1 in RWPE-1, DU145, and LNcap cells. **C** RIP assay combined with RT-qPCR analysis by EZH2 antibody for the binding to SChLAP1 in DU145 and LNcap cells with SChLAP1 knockdown. The results are presented as the mean ± SD. **P < 0.01, ***P < 0.001. SChLAP1 second chromosome locus associated with prostate-1, EZH2 Enhancer of Zeste Homolog 2, DNMT DNA methyltransferases.

To investigate the functional mechanism of SChLAP1 in regulating miRNA expression, we first test the potential involvements of methyltransferases in this process. By SChLAP1 probe RNA pull-down combined with western blot analysis, we showed that SChLAP1 may bind to EZH2 and DNMT3a protein in the prostatic cancerous cell DU145 (Fig. 2A). The direct interaction between EZH2/DNMT3a and SChLAP1 was further validated by RIP assay with EZH2/DNMT3a antibody. As the results show, we detected more enrichment of SChLAP1 with the EZH2 and DNMT3a in DU145 and LNcap cells compared to the RWPE-1 (human normal prostate epithelial cell) (Fig. 2B). Moreover, RIP assay results in SChLAP1-silenced DU145 and LNcap cells showed that the enrichment of SChLAP1 on EZH2 and DNMT3a were decreased (Fig. 2C). These results suggested that SChLAP1 could bind with EZH2 protein and DNMT3a.

### EZH2/H3K27me3 and DNMT3a may be the main reason for expression inhibition of multiple miRNAs on chromosome 5 in prostate cancer cells

**Fig. 3:**
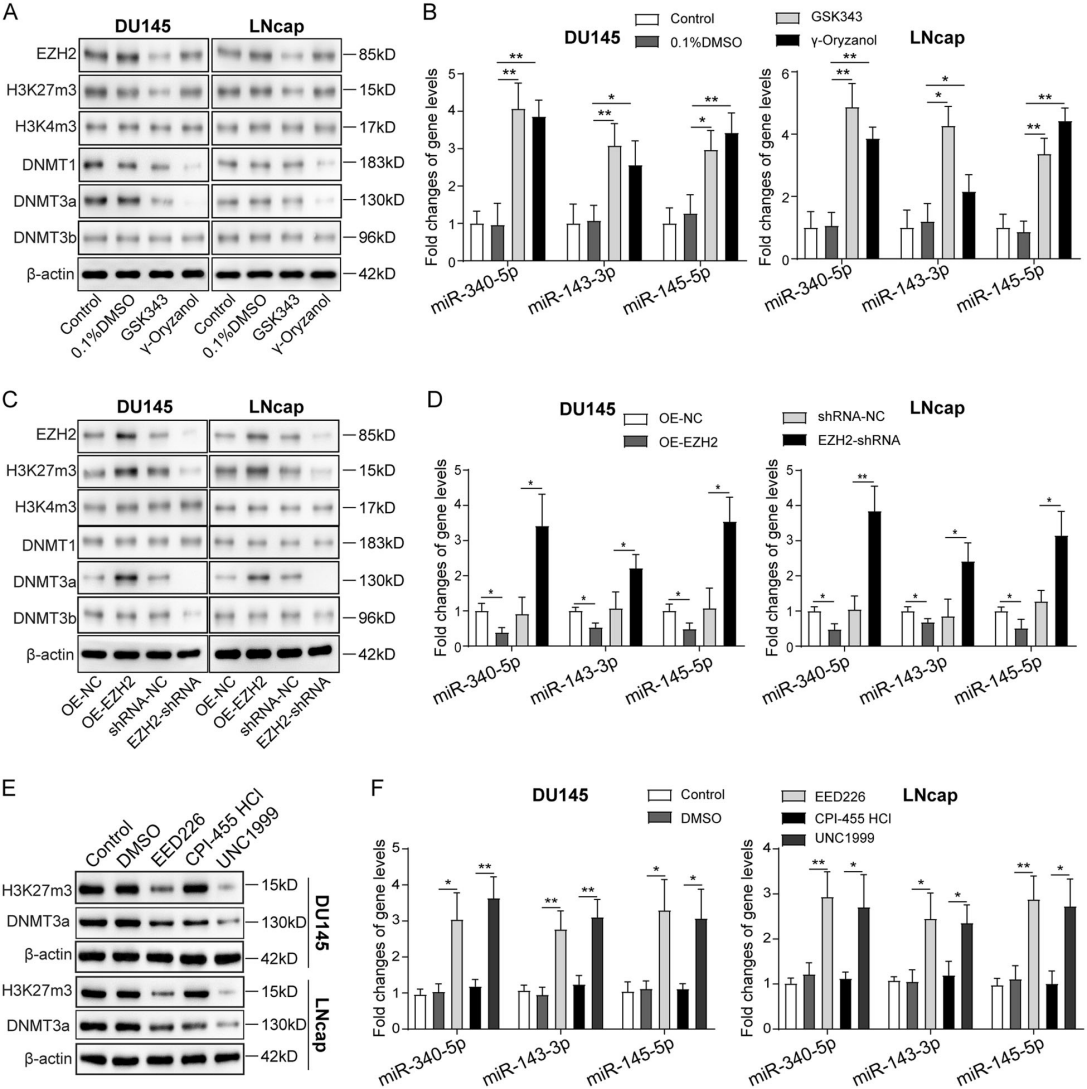
EZH2-mediated regulation of histone methylation may be the main reason for expression inhibition of multiple miRNAs on chromosome 5 in prostate cancer cells. **A** Western blotting analysis of EZH2, H3K27me3, H3K4me3, DNMT1, DNMT3a, and DNMT3b in the DU145 and LNcap cells treated with inhibitor GSK343 or gamma-Oryzanol or not. **B** Western blotting analysis of EZH2, H3K27me3, H3K4me3, DNMT1, DNMT3a, and DNMT3b in the DU145 and LNcap cells with EZH2 overexpression or knockdown. **C** Western blotting analysis of H3K27me3 in the DU145 and LNcap cells treated with EED226 or UNC1999 for H3K27me3 knockdown or treated with H3K4me3 inhibitor CPI-455 HCl. **D** QPCR analysis for the expression of miR-340-5p, miR-145-5p, and miR-143-3p in DU145 and LNcap cells treated with inhibitor GSK343 or gamma-Oryzanol or not. **E** QPCR analysis for the expression of miR-340-5p, miR-145-5p, and miR-143-3p in DU145 and LNcap cells with EZH2 overexpression or knockdown. **F** QPCR analysis for the expression of miR-340-5p, miR-145-5p, and miR-143-3p in DU145 and LNcap cells treated with EED226 or UNC1999 for H3K27me3 knockdown or treated with H3K4me3 inhibitor CPI-455 HCl. The results are presented as the mean ± SD. *P < 0.05, **P < 0.01. EZH2 Enhancer of Zeste Homolog 2, H3K27me3 histone 3 lysine 27 tri-methylation, H3K4me3 histone 3 lysine 4 tri-methylation, DNMT DNA methyltransferases.

For further exploration of the possible involvements of EZH2 and DNMT3a in regulating miR-340-5p, miR-145-5p, and miR-143-3p expression, we next inhibited EZH2 and DNMT3a activity in prostate cancer cells using specific inhibitors and gene knockdown technology. We found that EZH2 inhibitor GSK343 suppressed EZH2 and H3K27me3 levels in DU145 and LNcap cells, with no impacts on H3K4me3. Interestingly, the expression of DNMT3a, other than DNMT1 and DNMT3b, was significantly repressed by GSK343 in DU145 and LNcap cells, indicating that DNMT3a might serve a downstream target of EZH2. Meanwhile, γ-Oryzanol could specifically inhibit the expression of DNMT1 and DNMT3a, but not of DNMT3b, EZH2, H3K27me3, and H3K4me3. We observed that both GSK343 and γ-Oryzanol promoted miR-340-5p, miR-145-5p, and miR-143-3p expression in prostate cancer cells, showing the regulation of miR-340-5p, miR-145-5p and miR-143-3p expression by EZH2 and DNMT3a (Fig. 3A, B and Supplemental Fig. 1A). Moreover, EZH2 knockdown produced similar outcomes as GSK343 in prostate cancer cells, while EZH2 overexpression induced opposite alterations, which further validated the roles of EZH2 in regulating the miRNA expression (Fig. 3C, D and Supplemental Fig. 1B). In addition, H3K27me3 inhibitor EED226 or EZH2 inhibitor UNC1999 repressed H3K27me3 and DNMT3a and promoted the expression of these three miRNAs, which were not influenced by the H3K4me3-inducer CPI-455, further indicating that the expression of miR-340-5p, miR-145-5p, and miR-143-3p was mainly mediated by EZH2 and H3K27me3 other than H3K4me3 (Fig. 3E, F and Supplemental Fig. 1C).

### SChLAP1 recruits EZH2, H3K27me3, and DNMT3a to the promoters of miR-340-5p/miR-143-3p/miR-145-5p and represses their expression

**Fig. 4:**
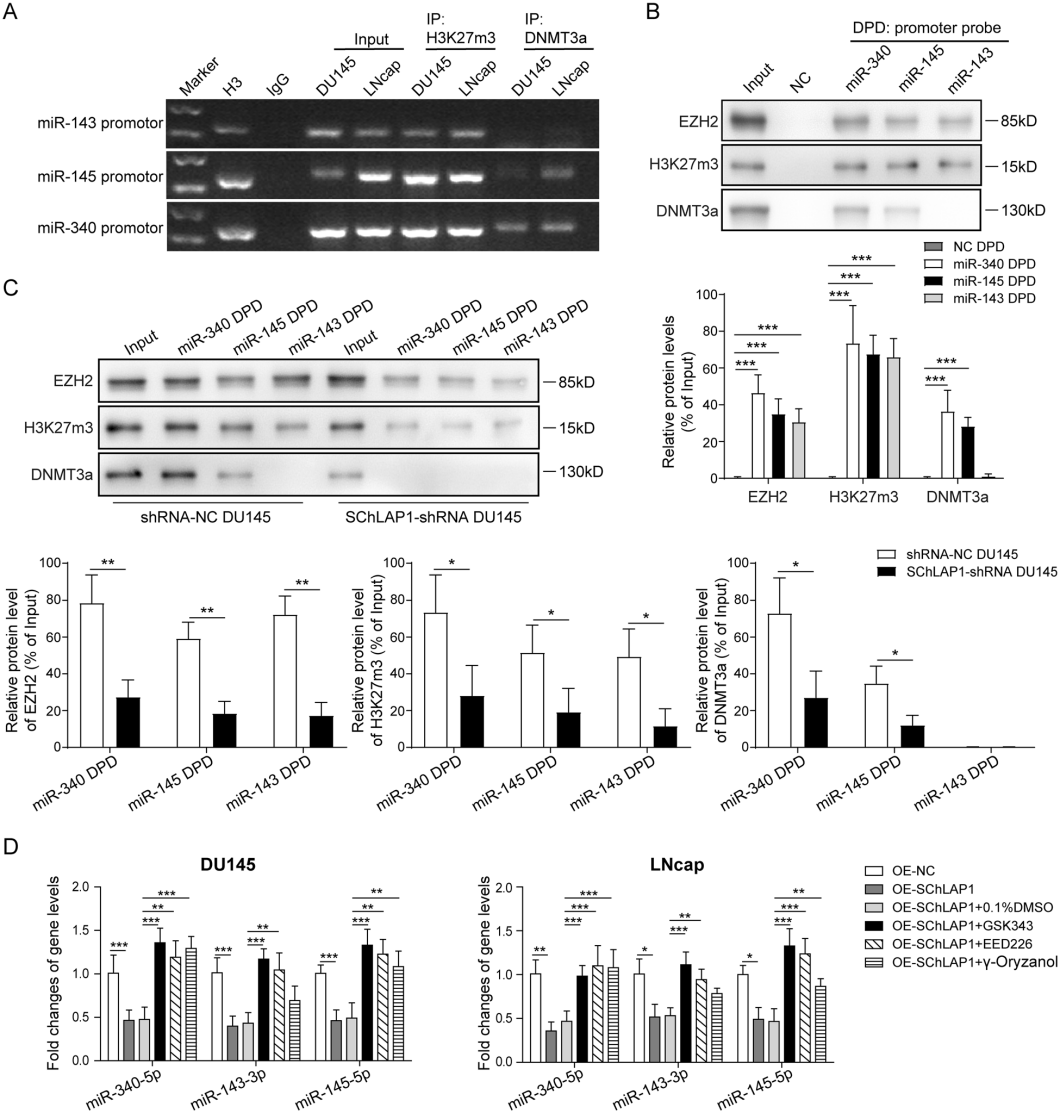
SChLAP/EZH2/H3K27me3 mediated promoter methylation modification of miR-340-5p/miR-143-3p/miR-145-5p to suppress gene transcription. **A** ChIP assay using anti-H3K27me3 and anti-DNMT3a antibodies for the interactions between H3K27me3 and DNMT3a with the promoter sequences of miR-340-5p/miR-143-3p/miR-145-5p genes. **B** DNA pull-down assay combined with western blotting analysis for the binding of EZH2, H3K27me3, and DNMT3a to miR-340-5p/miR-143-3p/miR-145-5p gene promoter, respectively, in DU145 cells using special DNA probes or negative control probe. **C** DNA pull-down assay combined with western blotting analysis for the binding of EZH2, H3K27me3, and DNMT3a to miR-340-5p/miR-143-3p/miR-145-5p gene promoter, respectively, in DU145 cell with SChLAP1 knockdown using special DNA probes. **D** Quantitative PCR assay for the effects of SChLAP overexpression, EZH2, H3K27me3, and DNMT3a inhibitors on miR-340-5p/miR-143-3p/miR-145-5p expression. The results are presented as the mean ± SD. *P < 0.05, **P < 0.01. SChLAP1 second chromosome locus associated with prostate-1, EZH2 Enhancer of Zeste Homolog 2, H3K27me3 histone 3 lysine 27 tri-methylation, DNMT DNA methyltransferases.

Base on the function mechanism of lncRNA/EZH2/H3K27me3 and lncRNA/DNMT3a-mediated gene inhibition, we subsequently tested the binding of EZH2, H3K27me3, and DNMT3a with miR-340-5p/miR-143-3p/miR-145-5p promoters by ChIP and DNA pull-down assays. Through ChIP assay using anti-H3K27me3 and anti-DNMT3a antibodies, results showed that H3K27me3 protein was closely bound with the promoter sequences of miR-340-5p, miR-143-3p, and miR-145-5p genes in DU145 and LNcap cells (Fig. 4A). Also, slight interactions between DNMT3a with the promoter sequences of miR-340-5p and miR-145-5p genes were observed, but no interaction between DNMT3a and miR-143-3p promoter was observed (Fig. 4A). Moreover, the following DNA pull-down assay using miRNA promoter probes further showed that the promoter sequences of miR-340-5p/miR-143-3p/miR-145-5p genes were bound with EZH2, H3K27me3, and DNMT3a proteins, except DNMT3a in pull-down production by miR-143-3p probe (Fig. 4B). When SChLAP1 was knocked down in DU145 cells, the binding interaction between EZH2/H3K27me3/DNMT3a and miRNA promoter sequences were dramatically attenuated compared to the negative control group (Fig. 4C), indicating that SChLAP1 could regulate the association of EZH2, H3K27me3, and DNMT3a with miR-340-5p/miR-143-3p/miR-145-5p promoters. Furthermore, SChLAP1 overexpression inhibited miR-340-5p/miR-143-3p/miR-145-5p expression in prostate cancer cells, which were abrogated by treatments with EZH2, H3K27me3, and DNMT3a inhibitors (Fig. 4D–F). Thus these results indicated that SChLAP1 inhibit the expression of miR-340-5p/miR-145-5p/miR-143-3p by recruitment of EZH2, H3K27me3, and DNMT3a to the promoter regions of three miRNA to mediate methylation modification (Figs. 1–4).

### DNMT3a was one of the common target genes of miR-340-5p/miR-143-3p/miR-145-5p in prostate cancer cells

**Fig. 5:**
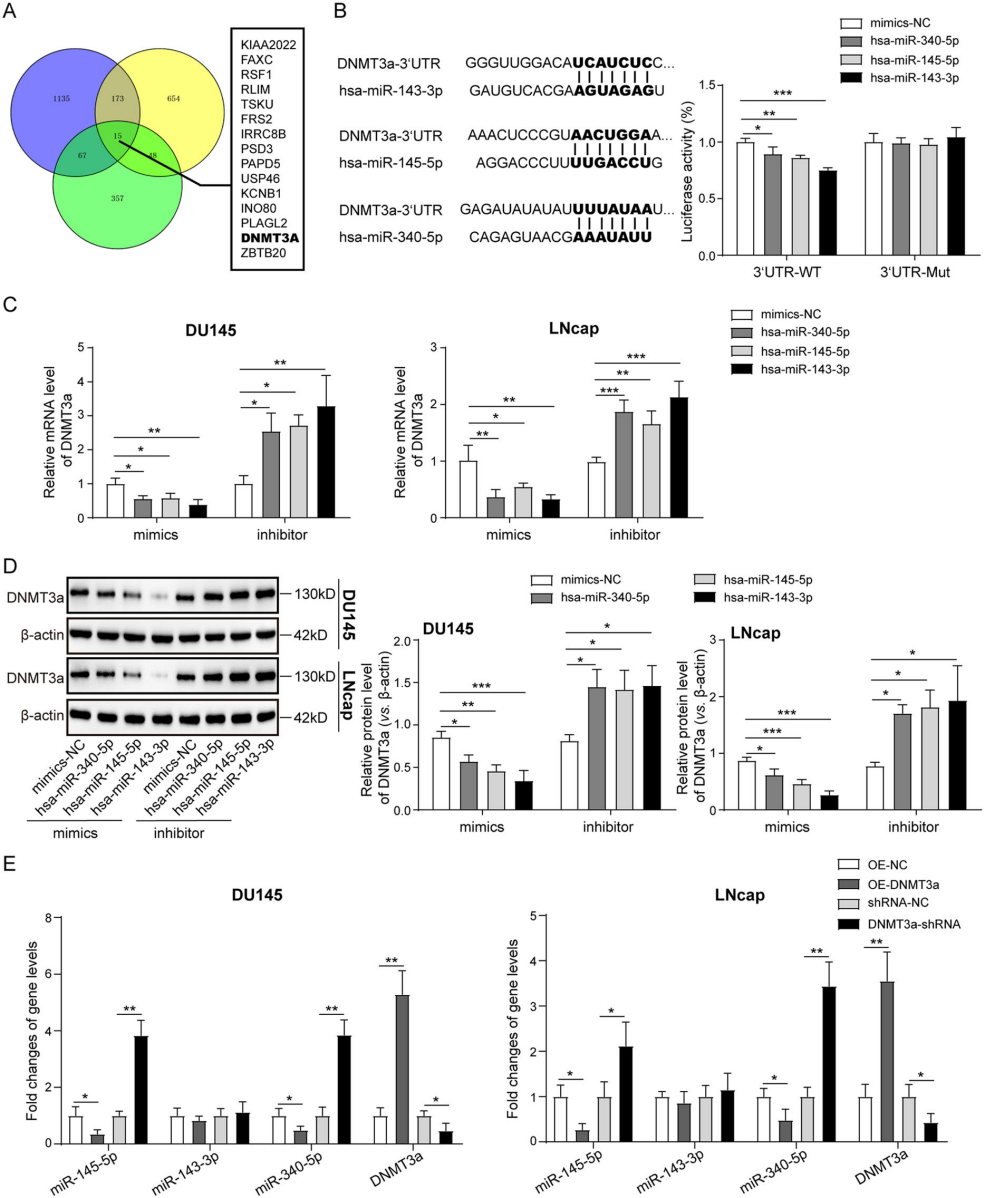
DNMT3a was one of the common target genes of miR-340-5p/miR-143-3p/miR-145-5p in prostate cancer cells. **A** Bioinformatic prediction analysis for the common target genes of miR-340-5p, miR-143-3p, and miR-145-5p using the online software TargetScan 7.2. **B** Dual-luciferase reporter assay for the verification of the direct targeted binding between DNMT3a and miR-340-5p/miR-143-3p/miR-145-5p. **C** QPCR analysis for the expression of DNMT3a mRNA level in DU145 and LNcap cells with miR-340-5p/miR-143-3p/miR-145-5p overexpression by miRNA mimics or knockdown by miRNA inhibitors. **D** Western blotting analysis for the expression of DNMT3a protein level in DU145 and LNcap cells with miR-340-5p/miR-143-3p/miR-145-5p overexpression by miRNA mimics or knockdown by miRNA inhibitors. **E** QPCR analysis for the expression of miR-340-5p, miR-143-3p, miR-145-5p, and DNMT3a mRNA in DU145 and LNcap cells with DNMT3a overexpression or knockdown. The results are presented as the mean ± SD. *P < 0.05, **P < 0.01. DNMT DNA methyltransferases.

Using bioinformatic prediction online software TargetScan 7.2, we found that there are 15 common target genes including DNMT3a for miR-340-5p, miR-143-3p, and miR-145-5p (Fig. 5A). Subsequently, dual-luciferase reporter assay was performed. Results showed that miR-340-5p, miR-143-3p, and miR-145-5p were all capable of directly binding with the 3’UTR sequence of DNMT3a mRNA to reduce the fusion luciferase activity (Fig. 5B), indicating that DNMT3a may be a common target gene of miR-340-5p/miR-143-3p/miR-145-5p. Furthermore, Overexpression of miR-340-5p/miR-143-3p/miR-145-5p by miRNA mimics in DU145 and LNcap cells greatly reduced the expression of DNMT3a mRNA and proteins, while knockdown of miR-340-5p/miR-143-3p/miR-145-5p by miRNA inhibitors markedly elevated the DNMT3a mRNA and protein expression in prostate cancer cells (Fig. 5C, D). Importantly, we further demonstrated that the expression of miR-340-5p and miR-145-5p in DU145 and LNcap cells were dramatically repressed by DNMT3a overexpression and elevated by DNMT3a shRNAs (Fig. 5E), indicating that there may be a feedback loop between DNMT3a and miR-340-5p/miR-145-5p, while miR-143-3p expression was unaffected by DNMT3a, consistent with the ChIP assay results that DNMT3a was not bound with the promoter region of miR-143-3p (Fig. 4A).

### SChLAP1/EZH2 promote prostate cancer cell proliferation and migration through the miR-340-5p/miR-145-5p-DNMT3a loop

**Fig. 6:**
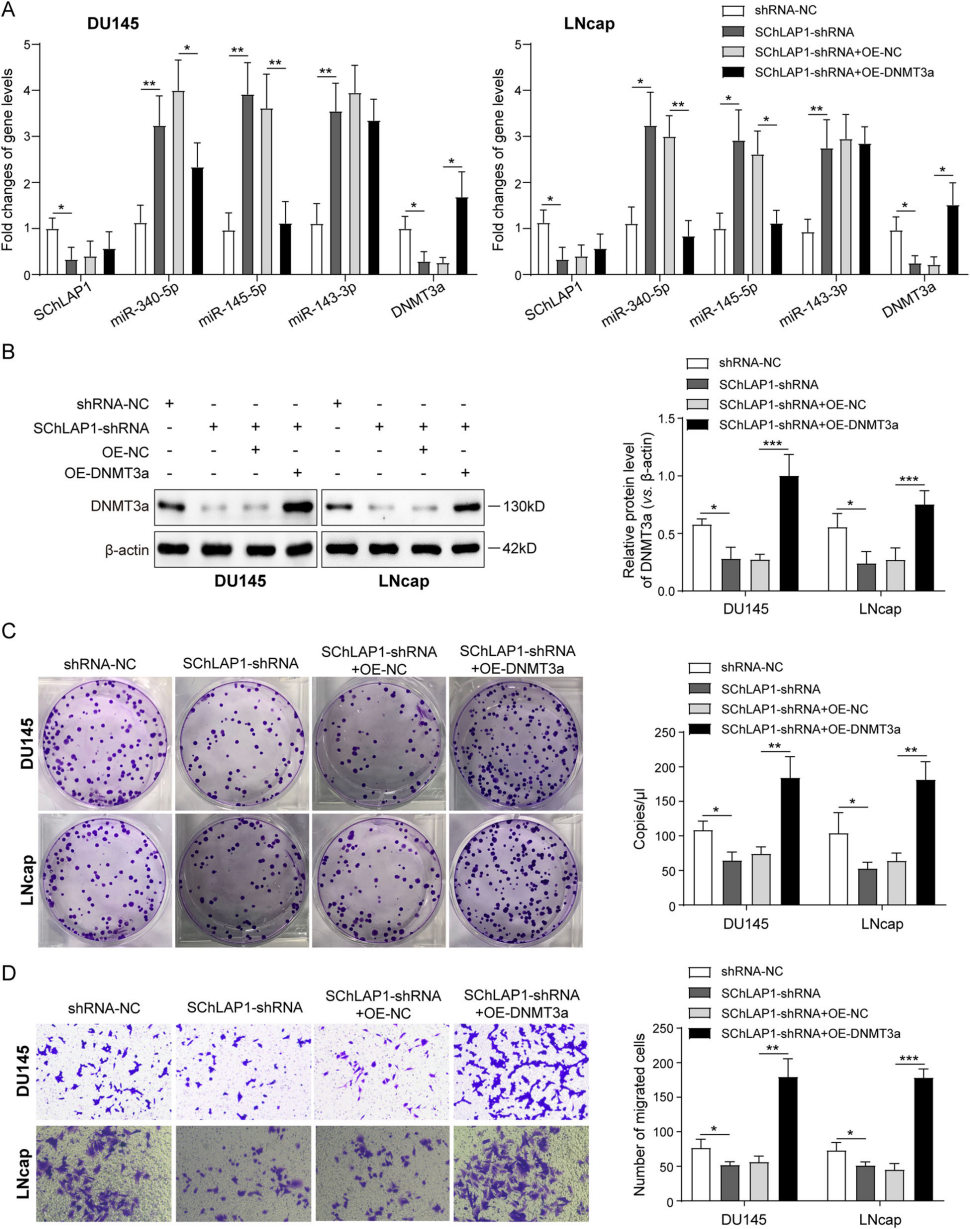
SChLAP1/EZH2 promote prostate cancer cell proliferation and migration through the miR-340-5p/miR-145-5p-DNMT3a loop. **A** QPCR analysis for the expression of SChLAP1, miR-340-5p, miR-143-3p, miR-145-5p, and DNMT3a mRNA in DU145 and LNcap cells with SChLAP1 silencing combined with DNMT3a expression or not. **B** Western blotting analysis for the DNMT3a protein level in DU145 and LNcap cells with SChLAP1 silencing combined with DNMT3a overexpression or not. **C** Colony-formation assay for cell proliferative capacity of DU145 and LNcap cells with SChLAP1 silencing combined with DNMT3a overexpression or not. **D** Transwell assay for cell migration capacity of DU145 and LNcap cells with SChLAP1 silencing combined with DNMT3a overexpression or not. The results are presented as the mean ± SD. *P < 0.05, **P < 0.01, ***P < 0.001. SChLAP1 second chromosome locus associated with prostate-1, EZH2 Enhancer of Zeste Homolog 2, DNMT DNA methyltransferases.

To explore the implication of above molecular events in prostate cancer development, we then tested their influences on prostate cancer cell functions. First, we observed that the silencing of SChLAP1-induced upregulation of miR-340-5p, miR-143-3p, and miR-145-5p expression and DNMT3a expression in DU145 and LNcap cells were significantly abrogated by the simultaneous overexpression of DNMT3a gene, except the miR-143-3p as expected (Fig. 6A, B). In addition, results of colony-formation assay for cell proliferative capacity showed that the SChLAP1 silencing greatly impaired the proliferation activity of DU145 and LNcap cells, which could be mitigated by DNMT3a overexpression (Fig. 6C). Similarly, the tumor migration capacity of DU145 and LNcap cells were significantly repressed by SChLAP1 knockdown, which was also completely abrogated by DNMT3a overexpression (Fig. 6D). So far, we proved here that SChLAP1 could effectively promote the proliferation and migration of prostate cancer cells, which was mediated by the above-discovered feedback loop between miR-340-5p/miR-145-5p and DNMT3a.

### SChLAP1/EZH2 promotes prostate cancer tumor development via the interaction of miRNA-DNMT3a signaling pathways in xenograft nude mice

**Fig. 7:**
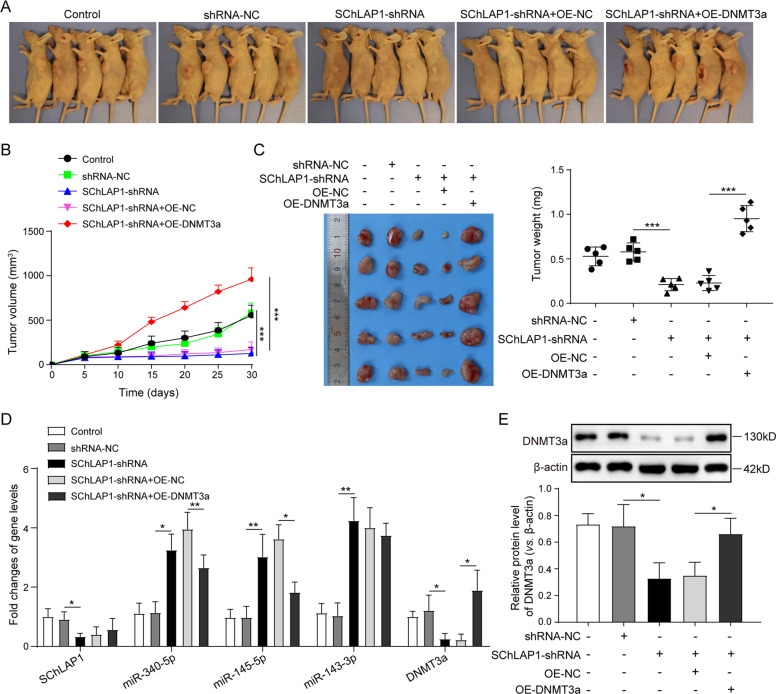
SChLAP1/EZH2 promotes prostate cancer tumor development via the interaction of microRNA-DNMT3a signaling pathways in xenograft nude mice. **A** Subcutaneous xenograft nude mice models injected with DU145 cells with stably expressed SChLAP1-shRNA and/or DNMT3a. **B** The volume of tumor growth in the nude mice injected with DU145 cells with stably expressed SChLAP1-shRNA and/or DNMT3a during 30 days. **C** The weight of tumors isolated from the nude mice injected with DU145 cells with stably expressed SChLAP1-shRNA and/or DNMT3a. **D** QPCR analysis for the expression of SChLAP1, miR-340-5p, miR-143-3p, miR-145-5p, and DNMT3a mRNA in tumors isolated from the nude mice injected with DU145 cells with stably expressed SChLAP1-shRNA and/or DNMT3a. **E** Western blotting analysis for the DNMT3a protein level in tumors isolated from the nude mice injected with DU145 cells with stably expressed SChLAP1-shRNA and/or DNMT3a. The results are presented as the mean ± SD. *P < 0.05, **P < 0.01, ***P < 0.001. SChLAP1 second chromosome locus associated with prostate-1, EZH2 Enhancer of Zeste Homolog 2, DNMT DNA methyltransferases.

For final validation of the function mechanism of SChLAP1/EZH2 in prostate cancer tumorigenesis, we established the CDX model by subcutaneous injection on nude mice with cultured prostate cancer cells. The volume and weight of tumors in nude mice injected with DU145 cells with stably expressed SChLAP1-shRNA were significantly smaller than that in the control group and shRNA-NC groups (Fig. 7A–C). However, DNMT3a overexpression substantially increased the tumor volumes and weight after 30 days growth (Fig. 7A–C). Moreover, the expression of DNMT3a mRNA and protein in tumor tissues was greatly repressed by SChLAP1 silencing, which resulted from great increases of miR-340-5p, miR-143-3p, and miR-145-5p expression in tumor tissues isolated from nude mice injected with SChLAP1-shRNA DU145 cells (Fig. 7D), whereas DNMT3a overexpression abrogated the above molecular alterations induced by SChLAP1 silencing in tumor tissues (Fig. 7D). Same changes were observed at the protein level of DNMT3a (Fig. 7E).

Together, these above results indicated that SChLAP2 promoted tumorigenesis of prostate cancer in vitro and in vivo through interacting with EZH2 to mediate a methylation-modification regulation in miR-340-5p/miR-143-3p/miR-145-5p and DNMT3a signaling pathways.

## Discussion

Epigenetic regulation has been established as essential mechanism underlying human cancer development and progression during the past decades^24^. The pathogenesis of prostate cancer were also shown to be mediated by epigenetic modulation^25^; however, the specific underlying epigenetic events remain poorly understood. In the present study, we characterized for the first time that the lncRNA SChLAP1, which was highly expressed in prostate cancer tissues and cell lines, promoted the development of prostate cancer through interacting with the histone methyltransferase EZH2 protein to repress the expression of multiple miRNAs, including miR-340-5p, miR-143-3p, and miR-145-5p. Moreover, we validated in this study that the roles of EZH2 protein in downregulating these miRNA expression in prostate cancer cells were mediated by its catalysis of the formation of H3K27 trimethylation (H3K27me3) located close to the promoters of genes encoding miR-340-5p, miR-143-3p, and miR-145-5p. Importantly, we proved that miR-340-5p, miR-143-3p, and miR-145-5p commonly target the expression of DNMT3a in prostate cancer cells, which also repressed the expression of these miRNAs as a feedback loop in prostate cancer cells. Finally, the roles of SChLAP1/EZH2 and the downstream miRNA-DNMT3a loop in prostate cancer pathogenesis were further validated by cellular and animal tumorigenic models. These investigations provided novel insights of prostate cancer development in terms of epigenetic regulation.

SChLAP1, also known as LINC00913, is one lncRNA that was highly expressed in prostate cancer cells^26^, which could independently predict metastasis and lethal progression in PC patients^27,28^. Previously, SChLAP1 was reported to promote PC development via inhibiting the chromatin-modifying SWI/SNF (SWItch/Sucrose Non-Fermentable) complex, which acts as chromatin remodeler and tumor suppressor^28^. Recent study showed that SChLAP1 performed its tumorigenic functions in a SWI/SNF-independent manner^29^. Moreover, SChLAP1 could enhance the proliferation and metastasis of PC cells through binding miR-198 to activate the MAPK signaling pathway^26^. However, the roles of SChLAP1 in regulating the expression of prostate cancer-suppressing miR-340-5p, miR-143-3p, and miR-145-5p in chromosome 5 remains unknown. We confirmed here the elevation of SChLAP1 expression in various prostate cancer cells, which was negatively correlated with the expression of miR-340-5p, miR-143-3p, and miR-145-5p. Furthermore, we demonstrated for the first time that SChLAP1 could directly bind with the EZH2 protein to repress miRNA expression through promoting H3K27me3 in prostate cancer cells, which established a new link between lncRNA, histone methylation, and miRNA expression associated with prostate cancer development.

Recent progresses by our group and other research teams showed that the suppression of miR-340-5p, miR-143-3p, and miR-145-5p expression encoded by #5 chromosome were critical for the initiation and progression of prostate cancer^10–14^. But little is known about the downstream target genes of these miRNA in prostate cancer development. DNA methylation catalyzed by DNA methyltransferases (DNMTs) are essential epigenetic events causing significant suppression of the expression of mRNAs and miRNAs as well^30^. Aberrant DNA methylation has been established as key molecular events contributing to tumorigenesis, and targeting DNMT1, DNMT3A, and DNMT3B with 5-azacytidine (Aza) and 5-Aza-2’-deoxycytidine were suggested as promising strategies for cancer treatment^31^. Through bioinformatics, we predicted DNMT3a as a common target gene of miR-340-5p, miR-143-3p, and miR-145-5p, which was further validated by our following dual-luciferase reporter assay combined with transfecting prostate cancer cell with miRNA mimics and inhibitors. These assays clearly proved that miR-340-5p, miR-143-3p, and miR-145-5p repressed DNMT3a gene expression via direct association with its promoter. On the other hand, DNMT1/3b-mediated DNA methylation also repressed the expression of multiple miRNAs in prostate cancer cells, thus forming a miRNA-DNMT1/3b feedback loop^12,15^. In this study, we also proved that DNMT3a could effectively repress the expression of miR-340-5p, miR-143-3p, and miR-145-5p in prostate cancer cells. Based on these results, we established a new feedback loop between DNMT3a and miR-340-5p/miR-143-3p/miR-145-5p underlying prostate cancer development. These results also suggested that the regulation of miRNAs encoded by #5 chromosome via DNMTs might be a prevalent epigenetic mechanism in prostate cancer.

To explore the pathogenic functions of SChLAP/DNMT3a in prostate cancer, we further evaluated their influences on prostate cancer cell proliferation, migration, and in vivo tumorigenicity using both the cellular and animal models. We clearly demonstrated here that the SChLAP1-shRNA repressed prostate cancer cell proliferation, migration, and tumorigenic capacities in nude mice, which was consistent with previous reports showing the oncogenic roles of SChLAP1 in prostate cancer pathogenesis^27,28^, although it has been suggested that the oncogenic functions of SChLAP1 was mediated by its modulation of the SWI/SNF complex and resultant chromatin remodeling^28^. However, later investigation clarified that the tumor-promoting effects of SChLAP1 in cancer cells was independent of SWI/SNF process^29^, and the specific molecular mechanisms underlying SChLAP1-regulated prostate cancer development still remains elusive. In this study, we demonstrated that the overexpression of DNMT3a effectively abrogated the SChLAP1 silencing-induced alterations of prostate cancer cell proliferation, migration, and tumor formation in nude mice, as well as miRNA expressional changes. These investigations convincingly proved the mediating roles of the miRNA-DNMT3a feedback loop in SChLAP1/EZH2-induced prostate cancer pathogenesis. In light of the prevalent and conserved roles of EZH2, DNMT3a, lncRNAs, and miRNAs in epigenetic regulation, the potential implications of the SChLAP1/EZH2/miRNA axis and the feedback loop between miRNAs and DNMTs in other human cancers also deserve further explorations.

**Fig. 8:**
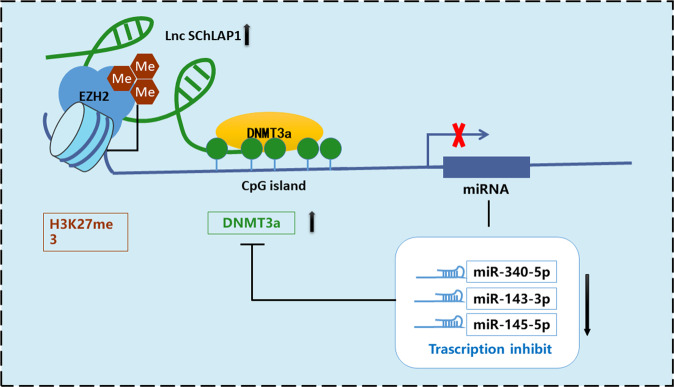
Schematic of the proposed mechanism of lncRNA SChLAP1 in prostate cancer. lncRNA SChLAP1 recruit EZH2 and DNMT3a to enhance H3K27me3 and DNA methylation, which will suppress the expression of miR-340-5p/miR-143-3p/miR-145-5p. DNMT3a is a common target gene of miR-340-5p/miR-143-3p/miR-145-5p. miR-340-5p/miR-143-3p/miR-145-5p was decreased while DNMT3a was increased in prostate cancer. There is a feedback loop between miR-340-5p/miR-143-3p/miR-145-5p and DNMT3a expression mediated the oncogenic roles of SChLAP1 in prostate cancer development.

In summary, we disclosed in this study that one new lncRNA SChLAP1, which was highly expressed in prostate cancer tissues and cell lines, promoted the proliferation, migration, and tumorigenicity of prostate cancer cells by interacting with EZH2 to enhance H3K27me3 and suppress miR-340-5p/miR-143-3p/miR-145-5p expression. Also, we proved here that the feedback loop between miR-340-5p/miR-143-3p/miR-145-5p and DNMT3a expression mediated the oncogenic roles of SChLAP1 in prostate cancer development (Fig. 8). These results provided novel insights into the epigenetic mechanisms associated with prostate cancer pathogenesis, which might be utilized as new biomarkers for prostate cancer diagnosis and drug development.

## Supplementary information

Supplementary Figure Legends

Figure S1
