# Supplementary material for: SChLAP1 promotes prostate cancer development through interacting with EZH2 to mediate promoter methylation modification of multiple miRNAs of chromosome 5 with a DNMT3a-feedback loop
Source: Cell Death Dis. 2021 Feb 15;12(2):188. doi: 10.1038/s41419-021-03455-8 (PMC7884413; doi:10.1038/s41419-021-03455-8)
Supplement: Supplementary file 1 — Supplementary Figure Legends [file 41419_2021_3455_MOESM1_ESM.docx]

Supplementary Figure 1 **The quantitative analysis of Figure 3 A, 3C and 3E**

(A) The quantitative analysis with the protein expression of EZH2, H3K27me3, H3K4me3, DNMT1, DNMT3a and DNMT3b in the DU145 and LNcap cells treated with inhibitor GSK343 or gamma-Oryzanol or not (the western blot result was show in figure 3A).

1. The quantitative analysis with the protein expression of EZH2, H3K27me3, H3K4me3, DNMT1, DNMT3a and DNMT3b in the DU145 and LNcap cells with EZH2 overexpression or knockdown (the western blot result was show in figure 3C).
2. The quantitative analysis with the protein expression of H3K27me3 in the DU145 and LNcap cells treated with EED226 or UNC1999 for H3K27me3 knockdown or treated with H3K4me3 inhibitor CPI-455 HCl (the western blot result was show in figure 3E).
