# Supplementary figures and images for: SChLAP1 promotes prostate cancer development through interacting with EZH2 to mediate promoter methylation modification of multiple miRNAs of chromosome 5 with a DNMT3a-feedback loop
Source: Cell Death Dis. 2021 Feb 15;12(2):188. doi: 10.1038/s41419-021-03455-8 (PMC7884413; doi:10.1038/s41419-021-03455-8)

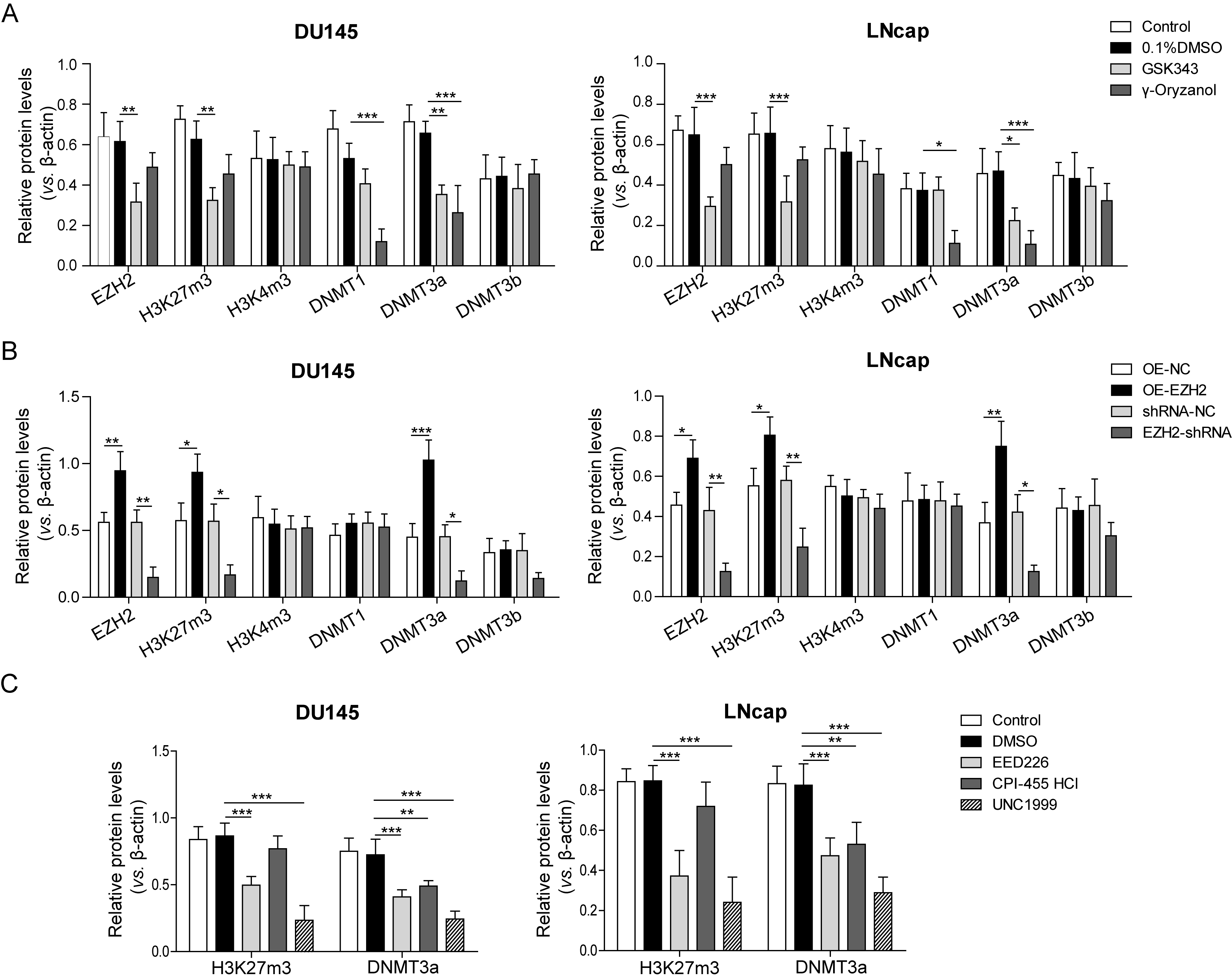

Supplement: Supplementary file 2 — Figure S1 [file 41419_2021_3455_MOESM2_ESM.tif]
